# Sterilizing Activity of Second-Line Regimens Containing TMC207 in a Murine Model of Tuberculosis

**DOI:** 10.1371/journal.pone.0017556

**Published:** 2011-03-03

**Authors:** Nicolas Veziris, Murad Ibrahim, Nacer Lounis, Koen Andries, Vincent Jarlier

**Affiliations:** 1 UPMC Université Paris 06, EA 1541, laboratoire de Bactériologie-Hygiène, Paris, France; 2 AP-HP, Hôpital Pitié-Salpêtrière, laboratoire de Bactériologie-Hygiène, Paris, France; 3 Centre National de Référence des Mycobactéries et de la Résistance des Mycobactéries aux Antituberculeux, Paris, France; 4 Department of Microbiology & Immunology, Faculty of Medicine, Al-Quds University Abu Dies, Jerusalem, Palestine; 5 Department of Antimicrobial Research, Tibotec BVBA, Beerse, Belgium; Statens Serum Institute, Denmark

## Abstract

**Rationale:**

The sterilizing activity of the regimen used to treat multidrug resistant tuberculosis (MDR TB) has not been studied in a mouse model.

**Objective and Methods:**

Swiss mice were intravenously inoculated with 6 log10 of *Mycobacterium tuberculosis* (TB) strain H37Rv, treated with second-line drug combinations with or without the diarylquinoline TMC207, and then followed without treatment for 3 more months to determine relapse rates (modified Cornell model).

**Measurements:**

Bactericidal efficacy was assessed by quantitative lung colony-forming unit (CFU) counts. Sterilizing efficacy was assessed by measuring bacteriological relapse rates 3 months after the end of treatment.

**Main Results:**

The relapse rate observed after 12 months treatment with the WHO recommended MDR TB regimen (amikacin, ethionamide, pyrazinamide and moxifloxacin) was equivalent to the relapse rate observed after 6 months treatment with the recommended drug susceptible TB regimen (rifampin, isoniazid and pyrazinamide). When TMC207 was added to this MDR TB regimen, the treatment duration needed to reach the same relapse rate dropped to 6 months. A similar relapse rate was also obtained with a 6-month completely oral regimen including TMC207, moxifloxacin and pyrazinamide but excluding both amikacin and ethionamide.

**Conclusions:**

In this murine model the duration of the WHO MDR TB treatment could be reduced to 12 months instead of the recommended 18–24 months. The inclusion of TMC207 in the WHO MDR TB treatment regimen has the potential to further shorten the treatment duration and at the same time to simplify treatment by eliminating the need to include an injectable aminoglycoside.

## Introduction

As the leading cause of death from curable infectious diseases worldwide, tuberculosis (TB) is a serious global health issue [1]. The high rates of TB incidence and prevalence in developing countries have a considerable impact on population-level morbidity and mortality, particularly in settings where HIV incidence rates are high [2]. Inappropriate administration of essential anti-TB drugs leads to the emergence of resistant bacilli, thereby creating an additional obstacle to TB control [3]. In 2004, the World Health Organization (WHO) reported more than 400,000 new cases of multi-drug resistant TB (MDR-TB), defined as being resistant to isoniazid and rifampin [4]. The WHO recommended regimen to treat MDR TB includes an injectable aminoglycoside, a fluoroquinolone, pyrazinamide and/or ethambutol if the strain is susceptible in addition to one or two less active second-line drugs (ethionamide, linezolid, PAS, cycloserine…). The recommended duration of treatment with this regimen is at least 18 months after sputum culture conversion and extended to 24 months in patients with extensive pulmonary damage [5]. The recommended duration of treatment for MDR TB patients has not been adequately studied in controlled clinical trials.

TMC207 (R207910) is a diarylquinoline with a new mechanism of action (inhibition of ATP synthase), and is in clinical development for treatment of both drug susceptible (DS) and MDR TB. Its minimal inhibitory concentration (MIC) against *Mycobacterium tuberculosis* (M.tb) is very low (0.06 µg/ml). In mice intravenously (IV) inoculated with M.tb, TMC207 combined with first-line anti-TB drugs shortened the treatment duration from 6 to 4 months [6]. In MDR TB patients, it increased the proportion of sputum culture conversion from 9 to 48% after 2 months of treatment [7]. The optimal second-line background regimen to combine with TMC207 and the duration of treatment with such regimen are not known. We conducted a study to determine the sterilizing efficacy and optimal duration of treatment of the WHO recommended MDR TB regimen with and without addition of TMC207. Part of the results presented here have been presented at the 48th ICAAC in Washington [8].

## Materials and Methods

The laboratory has been approved on July 27th, 2004 to carry out animal experiments. Nicolas Veziris who carried the animal experiments has the following license number: 75-1531. We followed the animal experiment guidelines of the Faculté de Médecine Pierre-et-Marie Curie.

### Antimicrobial Agents

TMC207 was provided by Johnson & Johnson (Beerse, Belgium), while the other compounds were purchased from the following manufactures: isoniazid (H) from Laphal (Allauch, France), rifampin (R), pyrazinamide (Z) from Aventis (Antony, France), moxifloxacin (M) from Bayer (Puteaux, France), amikacin from Bristol-Myers Squibb (La Défense, France), and ethionamide from Sigma (France).

### *Mycobacterium tuberculosis* strain

The H37Rv strain of M.tb was grown on Löwenstein-Jensen medium (LJ slants). Colonies were subcultured in Dubos broth (Becton, Dickinson and company, Le Pont de Claix, France) for 7 days at 37°C. The turbidity of resulting suspension was adjusted with normal saline to match that of standard 1 mg/ml suspension of *Mycobacterium bovis* BCG and was further diluted with normal saline to obtain a 0.2 mg/ml suspension for mouse inoculation.

### Infection of the mice

Four hundred seventy female four-week-old Swiss mice were purchased from the Janvier Breeding Center (Le Genest Saint-Isle, France). They were intravenously infected in the tail vein with 0.5 ml of bacterial suspension containing approximately 1.1×10^6^ colony forming units (CFU) of M.tb H37Rv.

### Chemotherapy

Mice were randomized into ten groups (table 1). Negative control group 1 consisted of 40 infected and untreated mice, of which 10 were killed the day after infection (D-18), 20 at beginning of treatment (D0), and 10 kept untreated for assessing mortality from tuberculosis. Group 2 was a positive control group with 40 mice receiving the standard WHO regimen for DS TB i.e. 2 months of rifampin (R) isoniazid (H) and pyrazinamide (Z) followed by 4 months of R and H (abbreviated as 2RHZ/4RH). In group 3, 30 mice received the most active TMC207 containing regimen described to date i.e. R, TMC 207 (J) and Z for 2 months followed by 2 months of J and R (2JRZ/2JR) [6]. In group 4, 90 mice received the WHO recommended MDR TB regimen: 2 months of amikacin (A), ethionamide (Et), moxifloxacin (M) and Z followed by EtM for 4, 7 or 10 months (2AEtMZ/4EtM, 2AEtMZ/7EtM, 2AEtMZ/10EtM). In group 5, 90 mice received JZM for 2 months followed by JM for 2, 4 or 7 months (2JZM/2JM, 2JZM/4JM, 2JZM/7JM). Group 6 contained 30 mice which were treated with the WHO MDR TB regimen to which J was added (2 JAEtMZ/4JEtM). Groups 7 and 8 contained 30 mice each receiving the MDR TB regimen for 6 months with J substituting for either M or Z (2JAEtZ/4JEt and 2JAEtM/4JEtM). Groups 9 and 10 contained 30 mice each, treated with either JM or JZ for 6 months (6JM and 6JZ).

**Table 1:** Experiment Design.

	Regimen	D-18	D0	4 mo	6 mo	7 mo	9 mo	12 mo	15 mo	Total
**1**	**Untreated**	10	20		10*					**40**
**2**	**2RHZ+4RH**				10		30			**40**
**3**	**2JRZ+2JR**			10		20				**30**
**4**	**2AEtMZ+4EtM**				10		20			**30**
	**2AEtMZ+7EtM**						10	20		**30**
	**2AEtMZ+10EtM**							10	20	**30**
**5**	**2JZM+2JM**			10		20				**30**
	**2JZM+4JM**				10		20			**30**
	**2JZM+7JM**						10	20		**30**
**6**	**2JAEtMZ+4JEtM**				10		20			**30**
**7**	**2JAEtZ+4JEt**				10		20			**30**
**8**	**2JAEtM+4JEtM**				10		20			**30**
**9**	**6JM**				10		20			**30**
**10**	**6JZ**				10		20			**30**
	**Total**	**10**	**20**	**20**	**100**	**40**	**210**	**50**	**20**	**440**

Definition of abbreviations: J = TMC 207, R = Rifampin, M = Moxifloxacin, H = Isoniazid, Z = Pyrazinamide, A = amikacin, Et = ethionamide.

Mice were infected intravenously with 1.1×10^6^ of *M. tuberculosis* H37Rv.

Day -18: the day after infection, Day 0: start of treatment.

*Mice kept untreated for mortality assessment.

All drugs were given 5 times per week at the following doses: J, 25 mg/kg; R, 10 mg/kg; M, 100 mg/kg; H, 25 mg/kg; Z, 150 mg/kg; A, 150 mg/kg; Et, 50 mg/kg.

Treatment began 19 days after intravenous infection (D0) i.e when a large bacterial population had developed. All groups were treated 5 days per week. Drug suspensions were prepared weekly and kept at 4°C except for TMC207 which was prepared monthly in 20% hydroxypropyl-B-cyclodextrin and kept at 4°C. Amikacin was given by subcutaneous injection at 150 mg/kg as previously described [9]. All the other drugs were given by gavage at the following doses: J 25 mg/kg, R 10 mg/kg, Z 150 mg/kg, M 100 mg/kg (once-a day), H 25 mg/kg and Et 50 mg/kg. R was administered one hour before other drugs to avoid drug-drug interactions [10], [11], [12]. Drug doses were selected to provide areas under the curve (AUC) values in mice comparable with those achievable in human [9], [11], [12], [13]. For consistency, 4 weeks of treatment was considered as corresponding to one month [11].

### Assessment of infection and treatment efficacy

To assess the extent of M.tb infection and to provide baseline values before initiation of chemotherapy, 10 untreated control mice were sacrificed at day 1 and 20 at day 19 after infection respectively (i.e. day −18 and day 0 in relation to the initiation of treatment). Treated mice were sacrificed either at the end of treatment to assess the bactericidal activity or 3 months after the end of treatment to assess the relapse rate (sterilizing activity) (Table 1).

The severity of infection and the effectiveness of the treatments were assessed by survival rate, spleen weight, gross lung lesions (0: no lesion, +: less than 10 tubercles, ++: more than 10 tubercles) and lung CFU counts.

The number of CFU in the lungs was determined by plating homogenized lung suspensions in triplicate on Lowenstein-Jensen (LJ) slants, either diluted for untreated control mice or undiluted for treated mice. In the latter case the whole suspension (3.5 ml) was plated on 15 LJ slants. The CFU count was assessed after 6 weeks of incubation at 37°C [14].

### Statistical analysis

Mean spleen weights were compared using the Student's t test. Proportion of positive mice 3 months after the end of treatment were compared using the Fisher's exact test (FET) to compare relapse rates since samples were small (less than 30 mice).

## Results

### Survival rate

Fify-two out of 440 mice died during the 15 months experiment. All of the 10 untreated mice died between 18 and 94 days after infection. Deaths among treated mice during the first month were assumed to be due to tuberculosis as mice appeared to be severely ill and had continuous weight loss after becoming infected whereas death occurring after the first month were due to gavage or injection accidents (such mice had all gained weight and splenomegaly had decreased). In group 2, 2 mice out of 39 died because of gavage accidents after the first month, in group 3, 2 mice out of 30 died (1 during first month and 1 afterwards), in group 4, 18 mice out of 90 died (6 during the first month and 11 because of gavage or injection accident afterwards), in group 5, 6 mice out of 90 died (5 during first month and one because of a gavage accident), in group 6, 4 mice out of 30 died (3 during first month and one because of gavage or injection accident), in group 7, no mice died, in group 8, 2 mice out of 30 died in the first month, in group 9, 3 mice died (2 during first month and one because of gavage accident) and in group 10, 6 mice out of 30 died (2 during the first month and 4 afterwards).

### Spleen weight

The day after infection (D-18), the mean spleen weight was 115±30 mg, while 18 days later, at the start of treatment (D0), the mean spleen weight had increased to reach 608±144 mg. During treatment, mean spleen weights decreased significantly in all the groups (p<0.005), compared to the mean spleen weight on D0 (results not shown).

### Gross lung lesions

The number of gross lung lesions increased from 0 the day after inoculation to ++18 days later at the start of treatment. After three and four months of treatment, the number of lesions decreased progressively. There was no difference in gross lung lesions among treated groups (results not shown).

### Enumeration of CFU in the lungs at the end of treatment

The day after infection, the mean lung CFU count was 5.6 log10. On D0, the mean lung CFU had increased to 7.1 log 10.

All mice were culture negative at the end of treatment in all treatment groups except 1 mouse in the JZM group after 4 months (3 colonies), 1 mouse in the AEtMZ group after 9 month (2 colonies) and 1 mouse in the AEtMZ group after 12 months (1 colony).

### Relapses after treatment completion

The proportion of mice that relapsed (i.e. became culture positive) 3 months after the end of treatment was determined for each combination (Table 2).

**Table 2:** Proportion of mice with positive culture of lung 3 months after treatment completion (relapse).*

Regimen	4 mo	6 mo	9 mo	12 mo
**2RHZ+4RH**		3/28 (11%)		
**2JRZ+2JR**	3/19 (16%)			
**2AEtMZ+4EtM**		11/19 (58%)^a^		
**2AEtMZ+7EtM**			8/16 (50%)^a^	
**2AEtMZ+10EtM**				4/18 (22%)^b^
**2JZM+2JM**	8/20 (40%)^a^			
**2JZM+4JM**		2/19 (11%)^b^		
**2JZM+7JM**			5/20 (25%)^b^	
**2JAEtMZ+4JEtM**		5/18 (28%)^b^		
**2JAEtZ+4JEt**		9/20 (45%)^a^		
**2JAEtM+4JEtM**		10/20 (50%)^a^		
**6JM**		9/20 (45%)		
**6JZ**		4/18 (22%)		

*Lung CFU count at start of treatment (D0): 7.1 log10 CFU.

Definition of abbreviations: J = TMC 207, R = Rifampin, M = Moxifloxacin, H = Isoniazid, Z = Pyrazinamide, A = amikacin, Et = ethionamide.

a significantly more relapses than RHZ (p<0.05).

b not significantly different from RHZ (p>0.05).

In the group receiving 2 months of RHZ followed by 4 months of RH, 3 out of 28 mice (11%) relapsed with a mean 1.8 log CFU. In the group receiving 2 months of JRZ followed by 2 months of JR, 3 out of 19 mice (16%) relapsed with a mean 2.5 log10 CFU. Among mice treated with the MDR WHO regimen, i.e. 2 months of AEtMZ followed by 4, 7 or 10 months of EtM, the relapse rates were 11/19 (58%) with a mean 2.4 log10 CFU, 8/16 (50%) with a mean 2.0 log10 CFU and 4/18 (22%) with a mean 0.9 log10 CFU. In the group receiving 2 months of JZM followed by 2, 4 or 7 months of JM, relapse rates were 8/20 (40%) with a mean 1.7 log10 CFU, 2/19 (11%) with a mean 0.3 log10 CFU and 5/20 (25%) with a mean 2.2 log10 CFU. Five out of 18 mice (28%) that received WHO MDR regimen combined with J, i.e. 2 months of JAEtMZ followed by 4 months of JEtM, relapsed with a mean 1.5 log10 CFU. Nine out of 20 mice (45%) that received the WHO MDR regimen in which J replaced M, i.e. 2 months of JAEtZ followed by 4 months of JEt, relapsed with a mean 2.3 log10 CFU. Ten out of 20 mice (50%) that received the WHO MDR regimen in which J replaced Z, i.e. 2 months of JAEtM followed by 4 months of JEtM, relapsed with a mean 1.8 log10 CFU. And finally, nine out of 20 mice (45%) that received the 6 months JM combination relapsed with a mean 1.8 log10 CFU and four out of 18 mice (22%) that received the 6 months JZ combination relapsed with a mean 0.9 log10 CFU.

Compared to the standard WHO DS regimen (group 2), the relapse rate of the WHO MDR regimen (AEtMZ, group 3) was significantly worse after 6 and 9 months (p = 0.0009 and 0.009 respectively) but not after 12 months (p = 1). When J was added to the WHO MDR regimen (group 6) the relapse after 6 months became equivalent to that of RHZ (p = 0.23). When M or Z were removed (groups 7 and 8) from this regimen the relapse rate increased again and became significantly worse compared to that of RHZ (p = 0.02 and 0.004, respectively).

Compared to the standard WHO regimen for DS TB (RHZ, group 2), the relapse rate of regimen JZM was significantly worse after 4 months (p = 0.03) but was equivalent after 6 and 9 months (p = 1 and 0.25, respectively). Even the regimen combining only J and Z (group 10) for 6 months achieved an acceptable relapse rate in this experiment.

## Discussion

We have previously shown that the MDR TB regimen combining amikacin, moxifloxacin, ethionamide and pyrazinamide has the highest bactericidal activity in the mouse model, resulting in culture conversion after 9 months of treatment [15]. However, in order to determine the minimal duration of treatment, one must conduct relapse studies studying the sterilizing activity of drug regimens [6], [16], [17], [18]. In the present work we studied the sterilizing activity of the WHO recommended regimen. The relapse rate obtained after 12 months was not statistically different from that obtained after 6 months treatment with the WHO recommended regimen to treat DS TB.

Several factors complicate the extrapolation of mouse data to the human situation. Firstly, the pathology of tuberculosis in mice is different from humans, the spread of the bacilli is haematogenous rather than bronchogenous, tuberculosis develops much faster than in humans (mice die in less than 2 months), and mice do not develop lung cavities [19]. For these reasons, we included the standard WHO treatment rifampin, isoniazid and pyrazinamide as a control group. Since the activity of this regimen is well established in man as well as in the murine model, it facilitates interpretation of the results [16].

Secondly, some dosages used here may not exactly mimic those used in humans. E.g. the 150 mg/kg dose of amikacin generates a Cmax in mice that is approximately four times higher than the one obtained in patients taking a 15 mg/kg dose. On the other hand, the intracellular penetration of aminoglycosides is known to be low and their efficacy is therefore expected to be better in patients with mainly extracellular TB replication compared to mice with mainly intracellularly replication [20]. Several studies in mice have indeed shown very limited activities of aminoglycosides, even when used at high concentrations [21], [22]. For moxifloxacin the 100 mg/kg dosing used in mice generates an AUC equivalent to the lowest AUC that can be achieved in patients and that is about 50% the highest AUC that can be achieved in patients [23], [24], [25]. The pyrazinamide dose is bioequivalent to that used in patients [16]. Taken together these comparisons suggest that the PK of the drugs used in this model are not far from the human PK at the usual dosing of these drugs. For ethionamide there are no mouse PK data allowing a comparison.

The third limitation is the statistical power of the study. To achieve a power of 80% to detect superiority between regimens, mouse relapse studies with 20 animals per group require an absolute difference of 40% in relapse rates between regimens [26], [27]. To achieve a power of 80% to demonstrate non-inferiority with an acceptable non-inferiority margin, the numbers of mice required in each group are much higher than those used in the current study. This means that two groups can have same relapse rate due to same activity or due to the fact that the difference of activity is too small to be revealed by the small number of animals. Consequently a lack of difference should be interpreted very carefully. Despite of this statistical limitation, it has been shown previously that the duration of DS TB treatment assessed in mouse relapse studies can be predictive for the duration of TB treatment in human [16]. The reproducibility and generalizability of this prediction will become clearer as more new TB regimens, now being tested in different mouse models, will be tested in clinical trials in the coming years.

The last limitation is that MDR TB isolates are by definition resistant to isoniazid and rifampin, but usually have also acquired additional resistance mutations. In this context, the impact of resistance to pyrazinamide on minimal treatment durations may be a very important one. Acquired pyrazinamide resistance is very common in MDR TB patients, and the data summarized in table 2 demonstrate that exclusion of pyrazinamide from a regimen leads to higher relapse rates (2JMZ+4JM 11% vs 6JM 45% p = 0.03 and 2JAEtMZ+4JEtM 28% vs 2JAEtM+4JEtM 50% p = 0.32). Similarly, resistance against fluoroquinolones is common in MDR TB patients and although our data do not suggest that such resistance would lead to higher relapse rates (2JMZ+4JM 11% vs 6JZ 22% p = 0.4 and 2JAEtMZ+4JEtM 28% vs 2JAEtZ+4JEtZ 45% p = 0.32), the addition of a third active drug to the combination of TMC207 and pyrazinamide is very important to prevent development of further resistance mutations. Our data should therefore not be seen as a rationale to shorten the recommended treatment duration for MDR TB patients. However this murine study supports the design of clinical trials assessing reduced treatment durations. A recent observational study by Van Deun and colleagues suggests that the duration of treatment for selected MDR TB patients can be reduced to 9 months using a clofazimine-based regimen [28]. Taken together the results of these two studies support the design of clinical trials evaluating the reduction of the treatment duration of the MDR-TB WHO recommended regimen from 18–24 to 12 months, if sensitivity to both moxifloxacin and pyrazinamide can be established [5].

The second aim of this study was to assess the contribution of the diarylquinoline TMC207 to the sterilizing activity of MDR regimens. TMC207 has been shown to improve both the bactericidal and the sterilizing activity of the first line regimen RHZ [6], [29]. The combination of TMC207 with the second-line MDR regimen (JAEtMZ) also resulted in acceleration of the bactericidal activity in a 2 months experiment [9]. In the present study the addition of TMC207 to the WHO MDR regimen (AEtMZ) shortened the time to achieve a relapse rate equivalent to that of RHZ from 12 to 6 months. Interestingly when amikacin and ethionamide were excluded from this combination, the relapse rate did not increase: the group receiving JZM for 6 months also achieved a relapse rate equivalent to that of RHZ after 6 months. This result is important since amikacin and ethionamide are not well tolerated in humans [30], [31] and it raises the possibility to use a completely oral regimen to treat MDR TB in a shorter time frame. Even the double combination of TMC207 and Z had surprisingly good sterilizing activity after just 6 months of treatment. As discussed above, double combinations of TB drugs are not warranted because of the risk of emergence of resistance.

The slight increase in relapse rate obtained by extending treatment with JM from 6 to 9 months (groups 5B and 5C) is puzzling. Such increase could be due to the fact that too few mice were studied per group (as discussed above in the limitations of the study), to the development of resistance, or to the possibility that the combination of TMC207+M is not active against bacilli persisting after 6 months of treatment. The emergence of resistance was excluded by testing the sensitivity of strains isolated after 9 months. The combination of TMC207+M might indeed not be sufficient to kill all persistent bacilli, but is very efficient if Z is added, and not stopped after 2 months. Indeed, it was recently reported that the regimen JZM resulted in acceptable relapse rates after just 5 months, and complete bacteriological cure (0% relapses) after 6 months of treatment [32]. These data suggest that pyrazinamide treatment is still efficacious after 2 months of treatment and suggests that the observed bactericidal synergy with J [33] is extended in the sterilizing phase.

The control group receiving the DS TB regimen JRZ achieved a relapse rate equivalent to that of the 6 months standard WHO regimen RHZ after 4 months. This confirms an earlier study [6] and suggests that substitution of isoniazid by TMC207 could shorten the treatment duration of DS TB to 4 months. A recent study also showed that substitution of isoniazid by TMC207 and rifampin by rifapentin led to an acceptable relapse rate after 3 months treatment [32].

In conclusion, we showed that treatment of mice with a moxifloxacin+pyrazinamide based regimen, as recommended by WHO for the treatment of MDR TB, results in stable cure in 12 months. An entirely oral regimen combining TMC207, moxifloxacin and pyrazinamide could reduce the duration needed to produce stable cure to only 6 months. These treatment duration estimates need to confirmed in clinical trials. Preliminary results of an ongoing clinical trial with TMC207 against MDR TB are encouraging [7], [34].
